# Profiles of HIV/AIDS stigma and psychological well-being in relation to embodiment among people living with HIV

**DOI:** 10.1007/s11136-025-04085-9

**Published:** 2025-10-23

**Authors:** Katarzyna Drabarek, Marcin Rzeszutek

**Affiliations:** Faculty of Psychology, University of Warsaw, Stawki 5/7, Warsaw, 00-183 Poland

**Keywords:** HIV/AIDS stigma, Psychological well-being, Embodiment, Latent profile analysis, Minority stress, People living with HIV (PLWH)

## Abstract

**Purpose:**

This study aimed to examine how profiles of perceived HIV/AIDS stigma and psychological well-being (PWB)—defined by positive/negative affect, life satisfaction, and health-related quality of life—relate to embodiment among people living with HIV (PLWH). It also explored the role of minority stress, particularly among sexual minorities, in shaping these experiences.

**Methods:**

A total of 540 PLWH completed validated measures of HIV/AIDS stigma, psychological well-being, embodiment, and minority stress. Latent profile analysis (LPA) was used to identify subgroups based on levels of stigma, PWB, and minority stress. Multivariate analyses examined differences in embodiment indicators across profiles, controlling for sociodemographic and medical variables.

**Results:**

Three distinct profiles emerged: (1) high stigma and low PWB,(2) low stigma and high PWB, and (3) average levels of all indicators. Embodiment levels differed significantly across profiles: Profile 2 showed the highest levels of positive embodiment, while Profile 1 reported the lowest. Minority stress was significantly higher in Lesbian, Gay, Bisexual, Transgender (LGBT) participants in Profile 1, supporting the concept of stigma accumulation.

**Conclusion:**

PLWH are a heterogeneous group with varying experiences of stigma, well-being, and embodiment. Greater psychological well-being and lower perceived stigma are associated with higher embodiment. LGBT PLWH face compounded minority stress, further impacting their embodiment and well-being. Findings highlight the importance of personcentered, intersectional approaches in stigma-reduction and mental health interventions for PLWH.

June 2021 highlighted the 40th anniversary of the first cases of human immunodeficiency virus (HIV) infection detected by the Centre for Disease Control (CDC) [1]. Over the decades, advances in treatment have transformed HIV from a terminal illness to a manageable chronic condition, with life expectancy for people living with HIV (PLWH) now comparable to the general population [2]. Despite this progress, PLWH continue to report lower psychological well-being than both the general population and individuals with other chronic illnesses, largely due to persistent stigma [3]. Although its explicit manifestations have changed, the overall impact remains significant and continues to be a major source of distress and a barrier to effective HIV care globally [4–6].

Despite a plethora of studies, HIV/AIDS stigma research lacks a unitary theoretical model that proposes mechanisms through which such stigma exerts its negative effects on PLWH life [5, 7]. In our study, we followed minority stress theory, which illustrates the uniqueness of stressors experienced by various stigmatized groups in society, including PLWH [8, 9]. More specifically, it was found that HIV/AIDS stigma may be much higher among sexual minorities who are HIV positive (Lesbian, Gay, Bisexual, Transgender (LGBT) community) [10, 11]. Thus, among PLWH, two levels of stigmatization are observed: one linked to being diagnosed with HIV (i.e., objective medical status defined by a specific social construction and reception) and the other related to being a sexual minority. It has been observed that PLWH from sexual minorities have even lower well-being and worse health than the general population of HIV/AIDS patients [8, 11, 12]. As a result, in PLWH belonging to the LGBT community, one may observe phenomena of *stigma accumulation* due to exposure to stigma-related stress associated with their double devalued social status [13].

Several authors have noted that PLWH are a very heterogeneous patient group with respect to coping with various aspects of living with HIV [13, 14]. Specifically, although they share the same medical diagnosis (i.e., HIV infection), PLWH display different trajectories in their psychological well-being (PWB) over time [11, 15]. Nevertheless, thus far, the dominant methodological attitude to the study of PWB in PLWH was a variable-centered approach, which disregards the problem of the heterogeneity of PWB indicators across individuals [16]. Consequently, applying the person-centered approach for exploring well-being outcomes among PLWH is increasingly recommended but still constitutes a research gap in HIV/AIDS context [14, 17].

The final novelty of our study is related to highly neglected variables in the HIV/AIDS stigma literature, namely PLWH’s body image [18, 19]. Starting from classic HIV/AIDS stigma theorists [20], several subsequent authors underscored the dual role of the body and its image in the process of the formation and maintenance of HIV/AIDS stigma [19, 21]. On the one hand, at the beginning of the HIV/AIDS pandemic, when there was no advanced treatment, bodily markers of HIV/AIDS were very visible signs of AIDS, which served as a direct source of discrimination experienced by PLWH [20]. On the other hand, although the advent of highly active antiretroviral therapy in 1996 substantially eliminated the visible bodily signs of HIV/AIDS, many PLWH still experience their bodies as generators of internalized stigma due to the irrational societal narratives regarding this illness, such as how HIV infection is transmitted [18, 21]. Furthermore, the great progress in HIV/AIDS treatment did not eradicate many of the debilitating side effects of treatment for PLWH, such as lipodystrophy syndrome or weight gain [18]. More specifically, a recent large epidemiological US study showed that more than half of PLWH experience emotional distress associated with their bodies, including changes in appearance and hair loss, and sexual issues, such as loss of interest or lack of sexual satisfaction [22]. Body image disturbances prevalent among PLWH are strongly linked to poorer psychological well-being, reduced treatment adherence, increased sexual risk behaviors, and lower quality of life [23]. In other words, it is impossible to fully understand the HIV/AIDS stigma process and the psychological well-being of PLWH while ignoring the issue of PLWH’s bodies; however, surprisingly, this variable is nearly absent from the respective literature.

Our research focuses on the broad construct of embodiment, which is grounded in philosophical writings of Merleau-Ponty and Foucault [24]. Merleau-Ponty [25, 26] viewed a body as the primary site of perceptual subjective engagement with the world and how it is perceived. Crossley [27], following Merleau-Ponty, wrote about embodiment as a central experience of positioning oneself in the world, through the body which is always actively engaged with its environment. This notion was further developed by Foucault’s perspective on the body as shaped and disciplined by social discourses and power relations [28]. Thus, embodiment offers a critical lens for understanding how stigma produced by societal narratives and inscribed through mechanisms of social control, becomes anchored in bodily experience, shaping how individuals perceive themselves and are perceived by others.

Embodiment in modern research goes beyond traditional body image by capturing the lived experience of the body as both positive and negative [29]. It includes body connection, comfort, agency, attuned self-care, bodily desires, and resistance to objectification [30]. Higher levels of embodiment are linked to lower body image disturbances [31] and are shaped by social factors like stigma, support, and cultural norms [32]. This marks a shift in body image research, from negative body image focused on appearance dissatisfaction to promoting positive body image (PBI), which includes body appreciation, acceptance, adaptive appearance investment, and protective information processing [33]. PBI research has informed well-being interventions, including for chronically ill populations [33].

The emphasis on positive embodiment becomes even more significant in the context of PLWH. While traditional studies have often emphasized body image struggles and deficits in chronic illness contexts, our approach explores how broader operationalizations of embodiment and thus PBI can enhance the understanding of the psychological well-being of PLWH.

## Current study

The aim of the current study was to examine the profiles of perceived HIV/AIDS stigma and psychological well-being (as operationalized by life satisfaction, positive and negative affects, and health-related quality of life) with regard to embodiment levels among PLWH. In addition, we controlled for the minority stress intensity related to two levels of potential stigmatization among participants: one related to being diagnosed with HIV only and the second related to being HIV infected and belonging to the LGBT community. We verified the following study hypotheses:

### Hypothesis 1

PLWH are heterogeneous in terms of psychological well-being, perceived HIV/AIDS stigma, and subjectively evaluated embodiment while controlling for selected sociomedical variables and minority stress intensity.

### Hypothesis 2

PLWH with higher levels of psychological well-being, a lower degree of perceived HIV stigma, and lower HIV-related minority stress experience higher embodiment levels while controlling for selected sociomedical variables.

### Hypothesis 3

PLWH with lower psychological well-being, a higher degree of perceived HIV stigma, and high HIV-related minority stress experience lower embodiment levels while controlling for selected sociomedical variables.

### Hypothesis 4

For PLWH representing the LGBT community, the perceived minority stress intensity will be higher, which may modify the strength of the relationships between the studied variables, as described in Hypotheses 2 and 3.

## Method

### Participants and procedure

The overall study sample comprised 540 participants aged 18-82 (*M* = 39.76; *SD* = 10.50). Participants were recruited consecutively during their routine visits to the Hospital for Infectious Diseases in Warsaw, the largest treatment facility for PLWH in Poland. Recruitment was conducted by the study authors through direct invitations in the waiting area. While no standardized formula for determining power in latent profile analysis (LPA) exists, published reviews and simulation studies suggest that samples of 300-500 participants are typically sufficient for stable and reliable profile estimation [34, 35]. Our final sample of 540 participants therefore meets these recommendations, supporting the robustness of the analyses.

Table 1 summarizes the sociodemographic and medical variables of the participants. The participants did not receive any remuneration for taking part in the study. Participation in the study was voluntary and required informed consent. The research proposal obtained approval from the local ethics committee. The participants filled out a paper-and-pencil version of the questionnaire in the hospital area with all the inventories and sociodemographic questions. The required time to complete the questionnaire was 20 min. The inclusion criteria were 18 years or more, HIV diagnosis, and undergoing antiretroviral treatment.

**Table 1:** Sociodemographic and clinical characteristics of the study sample (*N* = 540)

Variable	*N* (%)	Variable	*N* (%)
Gender		Sexual orientation	
Male	487 (90.2%)	Heterosexual	114 (21.1%)
Female	50 (9.3%)	Homosexual	364 (67.4%)
Other	1 (0.2%)	Bisexual	49 (9.1%)
Missing data	1 (0.2%)	Other	2 (0.4%)
Age in years (M ± SD)	39.76 ± 10.50	Missing data	11 (2.1%)
Education		Mode of HIV Transmission	
Primary	13 (2.4%)	Sexual transmission (male partner)	421 (78.0%)
Vocational	19 (3.5%)	Sexual transmission (female partner)	26 (4.8%)
Secondary	166 (30.7%)	Injecting drugs	25 (4.6%)
Higher	334 (61.9%)	Blood products	10 (1.9%)
Missing data	8 (1.5%)	Other	32 (5.9%)
In a stable relationship	264 (48.9%)	Missing data	26 (4.8%)
Stable employment	425 (78.7%)	Years of HIV (M ± SD)	11.18 ± 8.00
AIDS	58 (10.7%)	Years of ARV therapy (M ± SD)	8.55 ± 6.62
Detectable viral load	29 (5.4%)	CD4 level (M ± SD)	563.79 ± 287.59

M = mean; SD = standard deviation. ARV = antiretroviral therapy. CD4 count is presented in cells/µL. “Mode of HIV transmission” categories are based on participants’ self-reports. Missing data did not exceed 5% for any variable

### Measures

#### Satisfaction with life scale

Life satisfaction was assessed using the Satisfaction with Life Scale (SWLS) [36] in its Polish adaptation [37]. The SWLS includes five statements (e.g., “So far I have gotten important things I want in my life”) evaluated on a 7-point Likert scale, where 1 means “strongly disagree” and 7 means “strongly agree.” Higher scores indicate greater life satisfaction. The SWLS has been widely validated across various populations, demonstrating strong reliability and predictive validity for psychological well-being and overall quality of life [38].

#### Positive and negative affect schedule

Affect was assessed using the Positive and Negative Affect Schedule (PANAS-X) [39] in its Polish adaptation [40]. The PANAS-X comprises 20 descriptions of emotions: 10 items measure positive affect (PA) (e.g., “enthusiastic” and “excited”) and 10 items measure negative affect (NA) (e.g., “scared” and “nervous”). Participants rate their experience of each emotion during a specified timeframe on a scale from 1 (“not at all”) to 5 (“very strongly”).

#### WHO quality of life - BREF

Health-related quality of life (HRQoL) was assessed using the 26-item World Health Organization Quality of Life Brief Version (WHOQOL-BREF) [41] in its Polish adaptation. The WHOQOL-BREF is a self-administered instrument that evaluates quality of life across four domains: physical health, psychological health, social relationships, and environmental factors. Responses are provided on a 5-point scale, with higher scores indicating a better quality of life. The instrument is validated globally and is recommended for use in clinical populations. Domain scores were calculated based on WHO guidelines.

#### Berger HIV stigma scale

HIV-related stigma was assessed using the Berger HIV Stigma Scale [42] in its Polish adaptation [43]. The scale consists of 40 items covering four domains: personalized stigma, disclosure concerns, negative self-image, and concerns with public attitudes toward people with HIV. Some items overlap across domains, capturing nuanced experiences of stigma. Responses are provided on a 4-point Likert scale ranging from 1 (“strongly disagree”) to 4 (“strongly agree”). A total stigma score is calculated by summing item scores, with higher scores indicating greater stigma.

#### Minority status

Three potential minority statuses were analyzed: gender, sexual orientation, and AIDS diagnosis. Gender was categorized with three options: woman, man, and other. Sexual orientation was classified into four categories: heterosexual, homosexual, bisexual, and other. AIDS diagnosis was assessed as a binary variable (yes/no). These variables were included to capture intersectional influences on stigma and psychological well-being.

#### The sexual minority stress scale

The Sexual Minority Stress Scale (SMSS) was used to assess minority stress among the participants, focusing on stressors specific to sexual minorities. The SMSS is based on Meyer’s [9] minority stress model and includes several subscales that evaluate internalized homophobia, expectations of rejection, concealment, satisfaction with outness, and experiences of negative events related to sexual orientation. The Polish adaptation of the SMSS was used in this study [44]. Twenty-four items were adapted to fit the context of LGBTQ + and HIV discrimination.

#### The experience of embodiment

Embodiment was evaluated using the Polish adaptation of the Experience of Embodiment Scale (EES) [30]. The EES consists of six subscales that measure different facets of embodiment: positive body connection and comfort, body unencumbered adjustment, agency and functionality, experience and expression of sexual desire, attuned self-care, and resisting objectification. Each subscale includes multiple items rated on a 5-point Likert scale, with higher scores reflecting more positive embodiment experiences.

### Data analysis

All instruments demonstrated good to excellent internal consistency in the present study. The Satisfaction with Life Scale (SWLS) showed strong reliability (Cronbach’s α = 0.85). The Positive and Negative Affect Schedule (PANAS-X) demonstrated high internal consistency for both Positive Affect (α = 0.91) and Negative Affect (α = 0.91). The WHO Quality of Life - BREF exhibited excellent reliability for overall domain scores (α = 0.93). The Berger HIV Stigma Scale also demonstrated excellent internal consistency (α = 0.95), with subscale alphas ranging from 0.75 (Disclosure Concerns) to 0.95 (Personalized Stigma). Finally, the Experience of Embodiment Scale showed high reliability for the total score (α = 0.93), with subscale alphas ranging from 0.55 (Resisting Objectification) to 0.89 (Positive Body Connection and Comfort).

The data analysis consisted of three consecutive steps. First, we conducted latent profile analysis (LPA) [45] to extract subgroups differing in terms of HIV/AIDS stigma, components of psychological well-being (see PA/NA, life satisfaction, and HRQoL), and minority stress intensity. Next, we examined the differences between extracted subgroups in terms of embodiment levels while controlling for selected sociomedical data. Lastly, we investigated the above-mentioned associations between the studied variables among PLWH representing sexual minorities, who may experience elevated minority stress intensity. For the between-group comparisons, we conducted multivariate analysis of covariance (MANCOVA) and analysis of covariance (ANCOVA), followed by the Gabriel post hoc test, which was developed to compare groups of different sizes. The analysis was performed using Jamovi 2.3.28 and IBM SPSS Statistics 29.0 software.

## Results

Table 2 presents descriptive statistics for the analyzed variables: mean values, standard deviations, skewness, and kurtosis measures. None of the measures of skewness or kurtosis exceeded the value of 1 or − 1; therefore, a normal distribution of the analyzed variables was assumed.

**Table 2:** Descriptive statistics for analyzed variables

Variables	M	SD	min	max	S	K
1. Positive Affect	33.23	8.83	1	50	− 0.67	0.51
2. Negative Affect	21.39	8.90	3	47	0.63	− 0.33
3. Satisfaction with Life	20.50	6.45	4	35	− 0.27	− 0.31
4. Health Related Quality of Life	86.33	16.32	5	118	− 0.89	0.59
5. HIV/AIDS Stigma	77.52	28.16	2	160	− 0.28	0.44
6. Minority HIV Stress	4.04	4.11	0	21	0.70	0.49
7. Minority LGBT Stress	7.46	5.64	0	24	0.53	− 0.45
8. Positive Body Connection and Comfort	24.26	6.48	2	35	− 0.63	0.39
9. Body Unencumbered Adjustment	24.77	6.59	1	35	− 0.69	0.44
10. Agency and Functionality	22.06	4.92	4	30	− 0.72	0.70
11. Experience and Expression of Sexual Desire	13.66	4.17	1	20	− 0.56	− 0.01
12. Attuned Self− care	21.90	5.16	1	30	− 0.90	0.87
13. Resisting Objectification	12.55	3.37	1	20	− 0.19	0.12
14. Experience of embodiment	117.24	27.62	11	166	− 0.95	0.71

M = mean; SD = standard deviation; S = skewness; K = kurtosis. Embodiment variables are subscales of the Experience of Embodiment Scale: Positive Body Connection and Comfort, Body Unencumbered Adjustment, Agency and Functionality, Experience and Expression of Sexual Desire, Attuned Self-care, and Resisting Objectification. Higher scores reflect higher levels of the measured construct.

### Profiles of HIV/AIDS stigma, psychological well-being, and minority stress intensity

LPA was performed to extract subgroups of participants differing in terms of HIV/AIDS stigma, psychological well-being, and minority stress intensity. The values of the fit indices were examined to determine the number of subgroups with different profiles to extract. Table 3 depicts the values of fit indices for 4 solutions regarding the number of subgroups considered.

**Table 3:** Fit indices acquired for One, Two, Three, and four subgroups to extract in the course of latent profile analysis

	Number of subgroups			
	1	2	3	4
15. *AIC*	16. 13,428	17. 12,964	18. 12,832	19. 12,808
20. *AWE*	21. 13,576	22. 13,199	23. 13,155	24. 13,218
25. *BIC*	26. 13,473	27. 13,034	28. 12,929	29. 12,932
30. *CAIC*	31. 13,485	32. 13,053	33. 12,955	34. 12,965
35. *KIC*	36. 13,443	37. 12,986	38. 12,861	39. 12,844
40. Entropy	41. 1.0	42. 0.83	43. 0.80	44. 0.75

*AIC* = Akaike information criterion; *AWE* = approximate weight of evidence; *BIC* = Bayesian information criterion; *CAIC* = consistent Akaike’s information criterion; *KIC* = Kullback information criterion

Out of the five fit indices taken into consideration, all values were lower when comparing the solution based on three profiles to the solution based on two profiles. Moreover, the values of the approximate weight of evidence (AWE), Bayesian information criterion (BIC), and consistent Akaike’s information criterion (CAIC) were higher when comparing the solution based on four profiles to the solution based on three profiles. Since the lower values of fit indices indicate a better fit of the model, a solution based on extracting three subgroups with distinctive features was chosen.

**Fig. 1:**
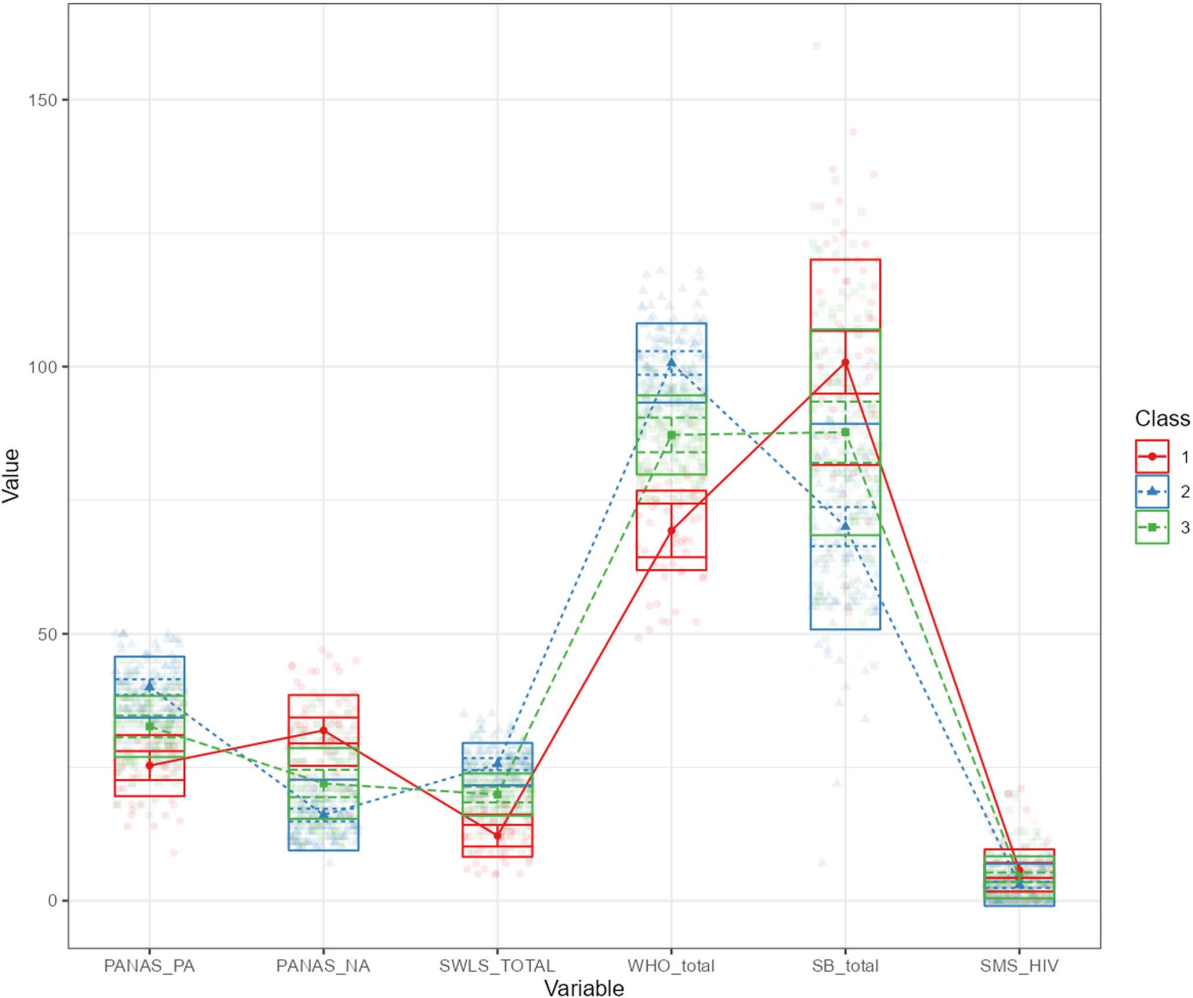
Profiles of HIV/AIDS stigma, psychological well-being, and minority stress intensity. Latent profile analysis identified three profiles among people living with HIV (PLWH): Profile 1 = high NA and stigma; Profile 2 = high PA and HRQoL; Profile 3 = intermediate levels across all variables. The Y-axis represents standardized scores for psychological well-being (PA,* NA*, life satisfaction, HRQoL), HIV stigma, and minority stress. PA = Positive Affect; NA = Negative Affect; HRQoL = Health-Related Quality of Life; SB = HIV/AIDS Related Stigma; SMS = Sexual Minority Stress

The first profile (*n* = 18.4%) was characterized by the highest level of HIV/AIDS stigma, the highest NA, and the lowest PA, as well as the poorest life satisfaction and HRQoL (see Fig. 1). The second profile (*n* = 38.2%) was characterized by the lowest levels of HIV/AIDS stigma and negative affect and the highest levels of positive affect, life satisfaction, and HRQoL. The values of all variables were acquired in the third profile (*n* = 43.4%). They were between the profile for subgroup 1 and the profile for subgroup 2. The acquired results support Hypothesis 1.

According to the value of the MANCOVA, the differences between the extracted profiles in terms of embodiment indicators were calculated when controlling for participants’ age, the length of HIV infection, being in a stable relationship, and participants’ education. All these associations were statistically significant, *F*(18, 492) = 10.51, *p* < 0.001, η^2^ = 0.28.

Table 4 presents the mean values of embodiment indicators in the three groups with three distinctive profiles of HIV/AIDS stigma, psychological well-being, and minority stress intensity, along with the values of analysis of variance and the results of post hoc comparisons performed with the use of the Gabriel post hoc test.

**Table 4:** Mean values of body image indicators in the groups extracted on the basis of psychological Well-Being, quality of Life, HIV Stigma, and minority stress

Profile Characteristics	Profile												
	No. 1		No. 2		No. 3						Post-hoc comparisons		
	NA + Stigma		PA + HRQoL		Average						*P*		
Variables	*M*	*SD*	*M*	*SD*	*M*	*SD*	*F*		*p*	η^2^	No. 1 vs. 2	No. 1 vs. 3	No. 2 vs. 3
45. Positive Body Connection and Comfort	18.58	5.06	28.69	4.69	24.14	4.84	81.15		0.001	0.39	0.001	0.001	0.001
46. Body Unencumbered Adjustment	20.26	5.02	28.64	5.48	25.00	5.69	46.44		0.001	0.27	0.001	0.001	0.001
47. Agency and Functionality	17.63	4.19	25.54	3.25	22.08	3.13	88.31		0.001	0.41	0.001	0.001	0.001
48. Experience and Expression of Sexual Desire	11.72	3.27	16.36	3.10	13.62	3.30	35.59		0.001	0.22	0.001	0.001	0.001
49. Attuned Self-care	18.91	4.15	25.09	3.07	22.43	4.33	39.54		0.001	0.24	0.001	0.001	0.001
50. Resisting Objectification	12.74	3.12	13.63	3.32	12.56	3.09	2.86		0.059	0.02	0.524	0.999	0.104
51. Experience of embodiment	99.84	14.48	137.71	17.61	119.84	17.22	97.25		0.001	0.44	0.001	0.001	0.001

*M* = mean value; *SD* = standard deviation; *F* = analysis of variance test; *p* = statistical significance; η^2^ = partial eta-squared effect size measure; PA = Positive Affect; NA = Negative Affect; HRQoL = Health Related Quality of Life; stigma = HIV/AIDS related stigma; Embodiment variables are subscales of the Experience of Embodiment Scale: Positive Body Connection and Comfort, Body Unencumbered Adjustment, Agency and Functionality, Experience and Expression of Sexual Desire, Attuned Self-care, and Resisting Objectification

The mean values of all indicators of embodiment, with the exception of resisting objectification, were significantly higher in the group of participants with Profile 2 than in the other two subgroups - Profile 1 or Profile 3. These results support H2. In addition, the mean values of all embodiment indicators, with the exception of resisting objectification, were significantly lower in the group of participants with Profile 1 than in the other two subgroups with Profile 2 or Profile 3. These results confirm H3.

Table 5 presents the mean values of the LGBT minority stress of participants with non-heterosexual orientation in the three groups with three distinctive profiles of HIV/AIDS stigma, psychological well-being, and minority stress intensity.

According to the value of ANCOVA, the differences between the three groups of participants with non-heterosexual orientation in terms of the level of LGBT minority stress were statistically significant when controlling for participants’ age, the length of HIV infection, being in a stable relationship, and participants’ education, *F*(2, 243) = 13.78, *p* < 0.001, η^2^ = 0.10. The level of LGBT minority stress was significantly higher in the group of participants with Profile 1 (i.e., with the highest level of HIV/AIDS stigma and negative affect and the lowest levels of positive affect, life satisfaction, and HRQoL levels) than in the other two subgroups with Profile 2, *t* = 5.40, *p* < 0.001, or Profile 3, *t* = 3.92, *p* < 0.001. The results obtained confirm H4.

**Table 5:** Mean values of LGBT minority stress in the groups extracted on the basis of psychological Well-Being, quality of Life, HIV Stigma, and minority stress

Profile	Profile characteristics	M	SD
52. No. 1	53. NA + Stigma	11.42	6.46
54. No. 2	55. PA + HRQoL	6.27	4.94
56. No. 3	57. average	7.70	5.14

M = mean; SD = standard deviation; NA = negative affect; PA = positive affect; HRQoL = Health Related Quality of Life; stigma = HIV/AIDS related stigma

## Discussion

The main aim of this study was to examine profiles of perceived HIV/AIDS stigma and psychological well-being with regard to embodiment levels among PLWH while considering the intensity of minority stress. The findings largely confirmed our hypotheses, shedding light on the heterogeneity of well-being among PLWH and the significant role of embodiment in this population. Our results supported Hypothesis 1, demonstrating heterogeneity in psychological well-being, HIV/AIDS stigma, and embodiment levels among PLWH. The LPA identified three distinct profiles. In Profile 1, the participants exhibited the highest levels of HIV/AIDS stigma and negative affect and the lowest levels of positive affect, life satisfaction, and HRQoL. The participants in Profile 2 showed the lowest levels of HIV/AIDS stigma and negative affect and the highest levels of positive affect, life satisfaction, and HRQoL. Profile 3 demonstrated moderate levels of all variables.

These findings highlight the variability in the psychological experience of PLWH and the necessity of a person-centered approach, as recommended by recent studies [46]. Unlike variable-centered methods, person-centered approaches allow for the identification of unique subgroups, offering more nuanced insights into how stigma and well-being manifest differently across individuals. These findings align with earlier research emphasizing the heterogeneity of PLWH’s experiences [11, 47, 48]. Studying the variability of PLWH experiences is crucial for the better development of patient-centered care, emphasizing tailored interventions informed by person-centered research that prioritize patients’ HRQoL and well-being [49].

Differences in embodiment emerged between the profiles. As hypothesized, PLWH with higher psychological well-being (Profile 2) exhibited higher levels of embodiment, while those with lower psychological well-being (Profile 1) reported lower embodiment levels. These results support Hypotheses 2 and 3 and underline the importance of the embodiment construct in HIV research. Similar findings have been reported in broader research exploring the role of PBI in psychological well-being among individuals facing chronic health challenges [50, 51]. Furthermore, as evidenced by Swami et al. [52], PBI is significantly associated with both hedonic and eudaimonic aspects of well-being.

Embodiment captures a broader spectrum of body-related experiences compared to traditional negative-oriented body image measures, as it encompasses positive body connection, agency, and attuned self-care [30]. These dimensions may be particularly relevant for PLWH, who often navigate complex relationships with their bodies due to HIV-related stigma, treatment side effects, and societal narratives [18]. Some patients experiencing lipodystrophy syndrome report a sense of alienation from their transformed bodies, with visible changes heightening stress and social vulnerability [53, 54].

The results of the study showed that the resisting objectification subscale in the EES did not show significant differences across profiles. This finding warrants further exploration. One possible explanation lies in the gender composition of our sample, which consisted of 90% men. Historically, research on objectification has focused on women’s experiences, as self-objectification theory was originally developed to address societal pressures and their impact on women [55, 56]. As accordingly to objectification theory, women’s bodies are socially constructed as objects of evaluation, which fosters self-objectification and habitual self-surveillance in anticipation of external judgment [55]. Research shows that women are women face heightened and more frequent appearance pressures compared to men [57]. This external orientation toward the body has been linked to lower body appreciation in women relative to men [58], and may contribute to difficulties in developing positive, embodied experiences.

However other studies [59] have suggested that men also experience objectification; the nature and intensity of this phenomenon differ by gender. While men generally face fewer consequences of objectification, sexual minority men (SMM) are more vulnerable to body image concerns due to being viewed through both masculine and sexualized lenses [60, 61]. In LGBT communities, physical appearance often equates to social capital, with lean, muscular bodies seen as desirable and healthy [62]. Due to this SMM face greater appearance pressures than heterosexual men and are more likely to report body dissatisfaction and engage in body-modifying behaviors, such as excessive exercise, dieting, and steroid use [63, 64]. These issues may be intensified among SMM living with HIV due to treatment-related bodily changes like lipodystrophy [18].

Moreover, the EES, while validated for use with men and women, was originally developed based on interviews with women and validated in female samples [30]. Since the development of the Embodied Experiences Scale (EES), it has been used in studies with mixed-gender samples across different countries, including Poland [65], Belgium [66], and Sweden [67], its applicability across diverse populations has been examined. Notably, the study by Kling et al. [67] focused on gender differences in embodiment while validating the instrument in a Swedish mix-gender sample. This study also reported low reliability for the Resisting Objectification (RO) subscale, particularly among men, a finding that aligns with the results of the present study. This consistency suggests that the RO subscale may not capture male experiences of objectification as effectively as it does for females. The gendered origins of the scale may limit its sensitivity to the distinct ways in which men experience or resist objectification, potentially affecting its validity in male populations. Therefore, our findings highlight the need for further research into gender differences in objectification and embodiment, within male-dominated samples, such as PLWH. Refining measurement tools to account for these differences could provide more accurate insights into how objectification impacts body-related experiences across genders. At the same time, existing literature points to pronounced gender differences in the experience of embodiment more broadly. The gender may interact with HIV-related stigma in unique ways, potentially amplifying it’s impact on embodiment and psychological well-being among women living with HIV. Future research should therefore explicitly investigate these gender-specific processes in PLWH to deepen the understanding of how stigma and embodiment unfold across genders.

Therefore, our findings highlight the need for further research into gender differences in objectification and embodiment, particularly within male-dominated samples, such as PLWH. Refining measurement tools to account for these differences could provide more accurate insights into how objectification impacts body-related experiences across genders.

Hypothesis 4 was confirmed, with LGBT PLWH in Profile 1 reporting significantly higher minority stress than those in Profiles 2 and 3. This supports minority stress theory [9] and the concept of stigma accumulation - where overlapping marginalized identities intensify stress [13, 68]. For sexual minority men, the intersection of HIV stigma and appearance pressures within gay communities may increase body surveillance, reduce body appreciation, and promote body-modifying behaviors [62]. Chronic exposure to stressors related to sexual orientation can further heighten body image concerns, especially in contexts where physical appearance is highly valued [63, 69]. Although our sample size did not allow for within-group analyses among LGBT participants, prior research shows that transgender individuals face distinctive body image concerns related to gender dysphoria and social visibility [70] while bisexual individuals may experience *double discrimination* from both heterosexual and gay communities [71]. Future research should examine these subgroup-specific processes to better understand their impact on embodiment and stigma.

Additionally, although the data were collected in a Warsaw-based clinic, it is one of the major HIV treatment centers in Poland and serves patients from across the country. Nevertheless, future research should examine whether living in larger cities versus rural areas affects perceived stigmatization, as differences in social context and access to support resources could meaningfully shape these experiences. LGBTQ people in rural areas in Poland face higher levels of social exclusion, and fewer community support networks compared to those in urban settings [72]. These disparities may exacerbate stigma and negatively impact embodiment among PLWH, underscoring the importance of considering place of residence in future studies.

Stigma accumulation reflects the interaction between general and relative minority statuses. For instance, gay men living with HIV often experience intensified internalized homophobia, while heterosexual women or non-heterosexual men with AIDS may face compounded disclosure stigma [68, 73, 74]. HIV stigma remains particularly pronounced among sexual and gender minorities, who are disproportionately affected by the epidemic [10]. These findings highlight the need for intersectional approaches to address the unique stressors faced by LGBT PLWH [75].

The study extends prior research by showing how minority stress contributes to reduced embodiment and well-being. Those in Profile 1 may face dual stigma, which is related to both HIV and sexual minority status, and resulting in higher distress and lower embodiment. Interventions addressing both HIV stigma and minority stress are essential for improving outcomes in this group [76, 77].

## Strengths and limitations

This study offers several strengths and limitations that warrant discussion. Among its strengths, it is one of the first to apply the construct of embodiment to HIV research, providing a multidimensional perspective on body-related experiences. Additionally, by employing LPA, the study captured the diversity of experiences among PLWH, offering a more individualized understanding of stigma and well-being. A further strength lies in its intersectional approach, accounting for minority stress and acknowledging the compounded challenges faced by LGBT PLWH.

However, the study also has limitations. This study has several limitations. First, our sample was predominantly male, which may limit the generalizability of the findings and calls for future research oversampling women to better capture gender-specific experiences of embodiment and stigma. Second, the sample size did not allow for meaningful within-group analyses among LGBT + participants. Future studies should recruit larger and more diverse LGBT + samples to examine subgroup differences (e.g., between gay, bisexual, and transgender individuals) and their potential impact on stigma, minority stress, and embodiment As the data were collected in a Warsaw-based clinic, and we did not assess participants’ place of residence, our sample may not fully reflect the experiences of PLWH from rural areas. Finally, the cross-sectional design limits causal inference, and longitudinal research is needed to further clarify the directionality of the observed associations. Its cross-sectional design restricts causal inferences, necessitating longitudinal research to explore the dynamics among stigma, embodiment, and well-being over time. The use of convenience sampling from a single hospital may limit the generalizability of the findings, and future studies should strive for more diverse samples. Finally, the lack of significant findings for the resisting objectification subscale highlights potential measurement gaps, suggesting a need for scale refinement to better address the unique experiences of PLWH.

## Conclusions

This study highlights the critical role of embodiment in understanding the psychological well-being of PLWH. By adopting a person-centered approach, we identified distinct profiles of stigma, well-being, and embodiment, underscoring the heterogeneity of experiences within this population. The findings emphasize the need for tailored interventions that address both the physical and psychological dimensions of living with HIV, particularly for LGBT PLWH who face compounded minority stress. Future research should continue to explore the intersection of stigma, embodiment, and well-being, leveraging longitudinal designs and diverse samples to build on these findings.
